# IL-4 receptors on human medulloblastoma tumours serve as a sensitive target for a circular permuted IL-4-*Pseudomonas* exotoxin fusion protein

**DOI:** 10.1038/sj.bjc.6600034

**Published:** 2002-01-21

**Authors:** B H Joshi, P Leland, J Silber, R J Kreitman, I Pastan, M Berger, R K Puri

**Affiliations:** Laboratory of Molecular Tumour Biology, Division of Cellular and Gene Therapies, Center for Biologics, Evaluation and Research, Food and Drug Administration, NIH Building 29B, Room 2NN10, 29 Lincoln Dr., Bethesda, Maryland, MD 20892, USA; Laboratory of Molecular Biology, National Cancer Institute, National Institutes of Health, Bethesda, Maryland, MD 20892, USA; Department of Neurological Surgery, University of Washington, Seattle, Washington, USA; Department Of Neurosurgery, University of California, San Francisco, California, USA

**Keywords:** medulloblastoma, IL-4 receptor, subunit composition, immunofluorescence

## Abstract

Cytotoxins directed to interleukin-4 receptors have shown to mediate relatively selective cytotoxicity against a variety of human cancer cells *in vitro* and *in vivo*. In an ongoing Phase I clinical trial, a recombinant protein comprised of circularly permuted IL-4 fused to a mutated form of *Pseudomonas* exotoxin (the fusion protein termed IL-4(38-37)-PE38KDEL or cpIL4-PE) has shown antitumour activity against malignant glioma. Human medulloblastomas are neuroectodermal tumours that occur in children and have a poor prognosis. The goal of this study was to determine whether human medulloblastoma derived cell lines express interleukin-4 receptor and whether interleukin-4 receptor expression is accompanied by sensitivity to cpIL4-PE. Medulloblastoma cell lines express interleukin-4 receptor at the protein and mRNA levels as determined by binding, indirect immunofluorescence and RT–PCR studies. These cells expressed IL-4Rα (also known as IL-4Rβ) and IL-13Rα1 (also known as IL-13Rα′) chains, however common γ_c_, a component of the interleukin-4 receptor system in immune cells was not detected. Consistent with the expression of IL-4R, cpIL4-PE was found to be highly and specifically cytotoxic to four of five medulloblastoma cell lines. Susceptibility of medulloblastoma cell lines to cpIL4-PE seemed to correlate closely to the functional IL-4 binding sites in general as demonstrated by ^125^I-IL-4 binding, but did not seem to correlate with mRNA or cell surface immunoreactive receptor protein expression. The sensitivity of medulloblastoma cells to cpIL4-PE could be eliminated by concurrent incubation with IL-4 or IL-13, but not with IL-2. None of these cell lines showed any change in proliferation upon treatment with exogenous IL-4. These studies establish the interleukin-4 receptor as a medulloblastoma-associated target for possible tumour-directed cancer therapy. Further studies are warranted to investigate interleukin-4 receptor expression in primary medulloblastoma tumours and sensitivity to cpIL-4PE *in vitro* and *in vivo*.

*British Journal of Cancer* (2002) **86**, 285–291. DOI: 10.1038/sj/bjc/6600034 www.bjcancer.com

© 2002 The Cancer Research Campaign

## 

Medulloblastomas are malignant brain tumours of neuroectodermal origin that arise in the cerebellum. They primarily occur in children within the first decade of life and carry a poor prognosis in the advanced stage of the disease (Stevens *et al*, 1991). New active agents are needed to improve survival and enhance the quality of life of these patients. Our previous studies have identified abundant expression of receptors for IL-4 (IL-4R) on human brain tumour cells (Husain *et al*, 1998; Joshi *et al*, 2000; Puri *et al*, 1994, 1996a). Based on our preclinical efficacy and safety studies, we have begun a Phase I clinical trial, in which a recombinant chimeric protein composed of circularly permuted IL-4 fused with *Pseudomonas* exotoxin (cpIL4-PE or IL4(38-37)-PE38KDEL) is administered intratumouraly in patients with high-grade glioma (Rand *et al*, 2000). Preliminary results suggest that cpIL4-PE given by this route is safe and has antitumour activity in some recurrent gliomas. Thus, medulloblastoma patients may benefit from targeted cancer therapeutics, which have low toxicity and high specificity.

In addition to demonstrating expression of IL-4R on cancer cells including brain tumour cells, we have extensively studied the structure and signal transduction of IL-4R on cancer cells (Kawakami *et al*, 2000; Leland *et al*, 2000; Murata *et al*, 1995, 1997a,b, 1998a,b; Obiri *et al*, 1993, 1997). We have shown that the IL-4R exists in three different types (Murata *et al*, 1995, 1998a). Type I (classical) IL-4Rs are composed of IL-4Rα (also known as IL-4Rβ) and IL-2Rγ chain (γ_c_), while type II (alternative) receptors are composed of IL-4Rα and IL-13Rα1 subunits (Kawakami *et al*, 2000; Leland *et al*, 2000; Murata *et al*, 1995, 1997a,b). In some cell types all three chains are present (Type III IL-4R). From reconstitution studies, we have observed that IL-4 forms a complex with only two chains of IL-4R at a time even when all three chains were transfected in CHO-K1 cells (Murata *et al*, 1998b). Whether all three chains form a productive IL-4R complex is still not known. We and others have shown that type I IL-4R are expressed in immune cells e.g., T and B lymphocytes, monocytes, and haematological malignancies (Puri, 1995). Type II IL-4R is expressed in the majority of solid tumour cells examined e.g., glioblastoma, breast carcinoma, AIDS-associated Kaposi's tumour, head and neck cancer, renal cell carcinoma and others (Murata *et al*, 1998a). Type III IL-4R is expressed predominantly in B cells and monocytes.

Besides the dissimilarity in structure between haematological and non-haematological cells, the signalling by IL-4R in these cells is also different. In immune cells, IL-4 primarily signals through Jak1 and Jak3 tyrosine kinases while in non-immune cells IL-4 signals through Jak1 and Jak2 tyrosine kinases (Murata *et al*, 1995, 1998a). Even though proximal signal transduction by different types of IL-4R is different, the distal signalling through signal transduction and activator of transcription (STAT) is the same. All types of IL-4R phosphorylate and activate STAT-6 protein (Murata *et al*, 1995, 1998a,b).

The significance of expression of IL-4R on solid tumour cells is not known. It is also not known whether human medulloblastoma cells express IL-4R and, if they do, which chains of these receptors are present on medulloblastoma tumour cells. In the present study, we have examined the mRNA and protein expression of various receptor subunits of IL-4R by RT–PCR, radiolabelled binding, and indirect immunofluorescence assays in different medulloblastoma cell lines. In addition, we have tested the cyototoxic effect of cpIL4-PE on various medulloblastoma cell lines.

## MATERIALS AND METHODS

### Medulloblastoma cell lines

UW-228-1, UW-228-2 and UW-228-3 cell lines were derived from a medulloblastoma tumour and generated at Washington University, Seattle, WA, USA (Keles *et al*, 1995). D283, and D341 cell lines were purchased from the American Type Culture Collection (ATCC), Manassas, VA, USA. All these cell lines grew as monolayers except D283, which grew mostly in suspension while D341 cell line grew as a suspension cell line. PM-RCC, renal cell carcinoma cell line was derived in our laboratory (Obiri *et al*, 1993) and H-9 T cells were purchased from ATCC. These cell lines were cultured in DMEM medium containing 10% foetal bovine serum (Biowhittaker, Walkersville, MD, USA), 1 mM HEPES, 1 mM non-essential amino acids, 100 units ml^−1^ penicillin, and 100 μg ml^−1^ streptomycin (Biowhittaker).

#### Recombinant cytokines and toxins

Recombinant circularly permuted IL4-toxin IL4(38-37)-PE38KDEL was produced and purified to >95% homogeneity as described previously (Kreitman *et al*, 1994, 1995b; Puri *et al*, 1996b). Recombinant IL-4 and IL-13 were produced as described (Kreitman *et al*, 1995a; Oshima *et al*, 2000).

### RNA extraction

Total RNA was extracted from medulloblastoma cell lines using the Trizol reagent (Gibco, Gaithersburg, MD, USA) as per manufacturer's instructions. Briefly, 5×10^6^ cells were pelleted and lysed with the reagent and centrifuged after adding chloroform. The RNA from aqueous phase was separated and precipitated with cold isopropanol. The pellet was washed with 70% ethanol twice, dried and reconstituted with RNase free water. RNA was quantitated after measuring the optical densities at 260 and 280 nm in a spectrophotometer and stored at −70°C.

### Reverse Transcriptase-Polymerase Chain Reaction (RT–PCR)

Levels of IL-4R subunit mRNA were determined by RT–PCR using primers and conditions previously described (Murata *et al*, 1997a).

### Immunofluorescence assay

Twenty thousand cells from different medulloblastoma cell lines were cultured in chambered glass slides (Lab Tek- Nalge Nunc International, Naperville, IL, USA) for 48 h as described (Joshi *et al*, 2000). The cells were washed with PBS and fixed in cold methanol : acetone (1 : 1) and incubated at −20°C for 2 h. The slides were then washed and rehydrated with PBS and used for indirect immunofluorescence analysis. Monoclonal antibody against IL-13Rα1 was purchased from Diaclone, Besancon, France. Mouse monoclonal antibody (M57) against IL-4Rα chain was a kind gift from Immunex Corporation, Seattle, WA, USA and polyclonal rabbit anti γ_c_ antibody was purchased from Santa Cruz, California, USA. Rehydrated cells in the chambered slide were incubated with 1% bovine serum albumin, 5% either goat or horse serum in PBS to block non-specific binding of antibody. The slides were washed and incubated either with the specified primary antibody (1 : 1500 for mAb IL-13Rα1, rabbit polyclonal and mAb for IL-4Rα) or isotype control (mouse IgG1 for IL-13Rα1 and IL-4Rα or rabbit IgG) for 2 h at room temperature. Slides were then washed three times with PBS for 5 min at room temperature and stained with secondary antibodies conjugated to either TRICT or FITC which were diluted in PBS containing 0.1% BSA as per manufacturer's recommendation. After three washes with PBS, slides were dried and layered with Vectashield anti-fluorescence fading mounting medium (Vector Laboratories, Burlingame, CA, USA) and cover slipped. The slides were viewed in a Nikon epifluorescence microscope using appropriate filters.

### ^125^I-IL-4 binding and displacement assay

IL-4 was iodinated with IODOGEN reagent (Pierce, Rockford, IL, USA) according to manufacturer's instructions. The specific activity of radiolabelled IL-4 was ∼22 μCi μg^−1^. The IL-4 binding assay was performed as previously described (Obiri *et al*, 1993; Puri *et al*, 1991). Briefly, tumour cells were harvested after brief incubation with versene (Biowhittaker, Walkersville, DE, USA), washed three times in Hanks balanced salt solution and resuspended in binding buffer (RPMI 1640 plus 1 mM HEPES and 0.2% human serum albumin). For the displacement assay, medulloblastoma cells (1×10^6^/100 μl) were incubated at 4°C with ^125^I-IL-4 (100–200 pM) with or without 200-fold molar excess of unlabelled IL-4. Following a 2 h incubation, cell bound radio-ligand was separated from unbound by centrifugation through a phthalate oil gradient and radioactivity determined with a gamma counter (Wallac). The number of receptors and binding affinities were determined as previously described (Obiri *et al*, 1993; Puri *et al*, 1991).

#### Protein synthesis inhibition assays

The cytotoxic activity of IL4-toxins was determined by protein synthesis inhibition assay as previously described (Puri *et al*, 1991). Protein synthesis inhibition directly correlates with cell death (Puri *et al*, 1996b). Typically, 10^4^ medulloblastoma cells were cultured in leucine-free medium with or without various concentrations of cpIL4-PE for 20–22 h at 37°C. For competition studies, cells were pre-incubated with IL-4 or IL-13 or IL-2 (2 μg ml^−1^) for 30 min at 37°C prior to the addition of cpIL4-PE to the cells. Then 1 μCi of ^3^H-Leucine (NEN Research Products, Wilmington, DE, USA) was added to each well and cells were incubated for an additional 4 h. Cells were harvested and radioactivity incorporated into cells was measured by a Beta plate counter (Wallac, Gaithersburg, MD, USA).

#### Cell proliferation assay

Proliferation assays were performed as described previously (Leland *et al*, 1995). Briefly, the cells were washed, and resuspended in CM in which the FBS content was reduced to 2%. 2×10^4^ cells for each cell line were plated in a 96-well plate and cultured for 6 h at 37°C in a 5% CO_2_ incubator. IL-4 (0.1–1000 ng ml^−1^) was added to the cultures and incubated further for 16 h. The cells were then pulsed with [^3^H]-thymidine (1 μCi well^−1^) for an additional 8 h and frozen at −70°C. The plates were thawed, harvested and radiolabel uptake was measured by a Beta plate counter (Wallac, Gaithersburg, MD, USA).

## RESULTS

### Expression of IL-4 binding sites and IL-4 receptor chains in medulloblastoma cell lines

By radiolabelled IL-4 binding assays, all five medulloblastoma cell lines tested were found to express varying numbers of IL-4 receptors (Table 1

**Table 1:**
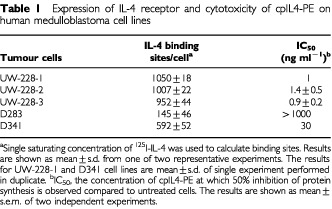
Expression of IL-4 receptor and cytotoxicity of cpIL4-PE on human medulloblastoma cell lines

). Subsequent RT–PCR analysis revealed that all five medulloblastoma cell lines expressed similar levels of mRNA for IL-4Rα and IL-13Rα1 chains, except D283 cell line which appear to show moderate expression for IL-13Rα1 chain as the intensity of mRNA band was lower compared to other cell lines (Table 2

**Table 2:**
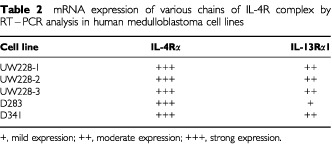
mRNA expression of various chains of IL-4R complex by RT–PCR analysis in human medulloblastoma cell lines

, Figure 1

**Figure 1:**
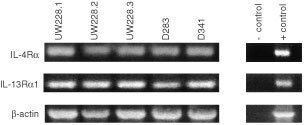
mRNA transcripts for IL-4R subunits. RT–PCR products were resolved in 2% agarose gel and visualized by EtBR staining. PM-RCC RNA served as positive control for IL-IL-13Rα1 and IL-4Rα chains.

). For a positive control, PM-RCC cells expressed mRNA for IL-4Rα and IL-13Rα1 chains. PM-RCC have been previously shown to express IL-4Rα and IL-13Rα1 chains (Obiri *et al*, 1997).

**Figure 2:**
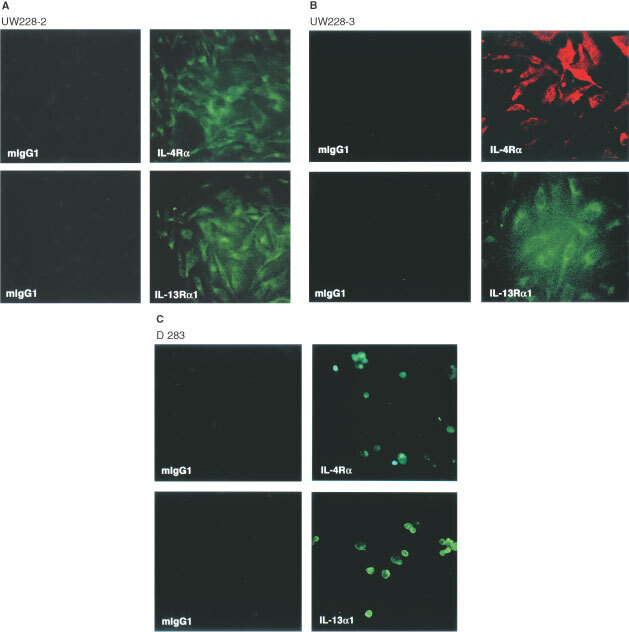
Immunofluorescence analysis of various receptor chains on medulloblastoma cell lines. (**A**) UW228-2, (**B**) UW228-3, and (**C**) D283 medulloblastoma cell lines were stained with either murine IgG1 isotype control or mouse monoclonal antibody to IL-4Rα chain or IL-13Rα1 chains. D283 cell line grew as a suspension culture showing few cells positive in the microscopic field.

We next examined the expression of various IL-4R receptor chains by indirect immunofluorescence assays in five different medulloblastoma cells lines. As shown in Figure 2

, immunofluorescence staining for IL-4Rα and IL-13Rα1 chains was very intense in UW228-2, UW228-3 and D283 cell lines. In contrast, the immunofluorescence staining of cells with matched isotype control antibody did not reveal any staining at all. Interestingly, D283 cell line showed strong immunoreactivity with a small number of cells even though the microscopic field shown had more cells. These results indicate that IL-4R expression is heterogeneous and all cells do not express IL-4R. Similar IL-4R staining results were observed in UW228-1 and D341 cell lines (data not shown). In contrast, IL-2Rγ_c_ protein was not detected in all five medulloblastoma cell lines tested (data not shown).

### Medulloblastoma cell lines are highly sensitive to the cytotoxic effect of cpIL4-PE

To determine whether IL-4R on medulloblastoma cell lines could be used as a therapeutic target, we tested sensitivity of these cells to cpIL4-PE. CpIL4-PE is a chimeric protein in which amino acids 38–129 of IL-4 were fused via a peptide linker to amino acids 1–37, which are in turn fused to amino acids 353–364 and 381–608 of PE, with KDEL at positions 609–612 (Kreitman *et al*, 1994, 1995b; Puri *et al*, 1996b). This purified fusion protein was found to be highly and specifically cytotoxic to a variety of human solid tumour cell lines including glioblastoma multiforme (GBM) *in vitro* (Puri *et al*, 1996a,b). We have also shown that this cytotoxin is highly active in the regression of established human xenografts in nude mice. Thus far, cpIL4-PE has been tested against epidermoid, GBM, AIDS-KS, and breast tumour models (Husain *et al*, 1998, 1999; Kreitman *et al*, 1995b; Leland *et al*, 2000). Remarkable antitumour activity including complete disappearance of established disease has been observed. As *in vitro* sensitivity to cpIL4-PE seems to correlate with IL-4R expression and *in vivo* antitumour activity and medulloblastoma cells expressed IL-4R, we tested whether cpIL4-PE is also cytotoxic to medulloblastoma cell lines.

**Figure 3:**
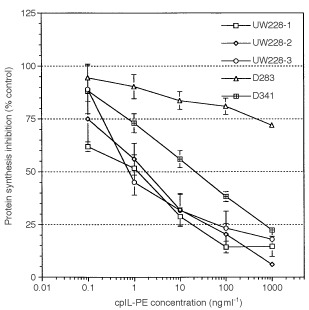
Cytotoxicity of cpIL-4-PE against medullobalstoma cell lines. Ten thousand cells were cultured with various concentrations of cpIL4-PE. Cells were then pulsed with 1 μCi [^3^H]-Leucine and cell-associated radioactivity was measured with a Beta Plate Counter. The results are shown as mean ±s.d. of quadruplicate determinations and the experiment was repeated two times.

**Figure 4:**
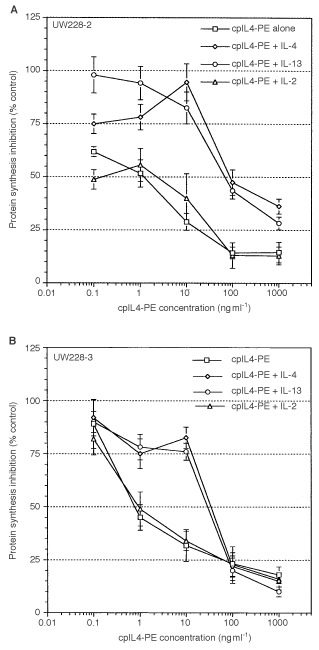
Both IL-4 and IL-13, but not IL-2 neutralize the cytotoxic activity of cpIL4-PE: (**A**) UW-228-2 and (**B**) UW228-3 cells (10^4^) were cultured with various concentrations of cpIL4-PE. For blocking experiments, cells were pre-incubated with IL-2, IL-4 or IL-13 (2 μg ml^−1^) for 30 min prior to the addition of cpIL4-PE. Data are shown as mean ±s.d. of quadruplicate determination.

As shown in Figure 3

and Table 1, three of five medulloblastoma cell lines expressing >900 IL-4 binding sites/cell were highly sensitive to the cytotoxic activity of cpIL4-PE (IC_50_=1 ng ml^−1^). While one cell line (D341), which expressed ∼600 sites/cell was moderately sensitive (IC_50_=30 ng ml^−1^) and one cell line (D283), which expressed the lowest level of IL-4R, was not sensitive to cpIL4-PE at concentrations up to 1000 ng ml^−1^. Thus, cpIL4-PE induced cytotoxicity positively correlated with IL-4 binding sites/cell. The cytotoxic activity of cpIL4-PE was neutralized by addition of excess IL-4 and also by IL-13 in UW-228-2 and UW-228-3 cell lines. As excess of IL-2 did not neutralize the cytotoxicity of cpIL4-PE, these results indicate IL-4R are related to IL-13R on medulloblastoma cell lines and cytotoxicity mediated by cpIL-4PE is somewhat specific (Figure 4

). This property of sharing of receptors for IL-4 and IL-13 has also been observed in other cancer cell lines and thus appears to be universal (Kawakami *et al*, 2000; Leland *et al*, 2000; Murata *et al*, 1997b).

### IL-4 does not modulate growth of medulloblastoma cell lines

**Figure 5:**
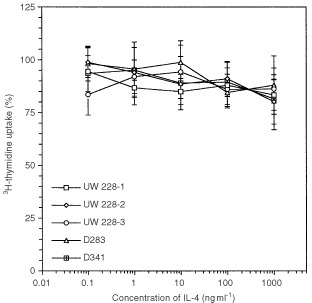
Exogenous IL-4 does not modify the growth of medulloblastoma cells: 2×10^4^ cells of five medulloblastoma cell lines were grown in the absence or presence of 0.1 ng to 1000 ng ml^−1^ IL-4. The cells were pulsed with 1 μCi of [^3^H]-thymidine and harvested after 8 h. The incorporated radiolabelled thymidine was measured and shown as per cent of control. Data are shown as mean ±s.d. of quadruplicate determinations.

Since IL-4 has been shown to modulate growth of various tumour cell lines (Puri, 1995), we examined whether it modulated growth of human medulloblastoma cell lines. As shown in Figure 5

, IL-4 did not alter [^3^H]-thymidine incorporation in any cell line tested even with IL-4 up to a concentration of 1000 ng ml^−1^. These results indicate that IL-4 does not modulate growth of medulloblastoma cell lines.

## DISCUSSION

We demonstrate that medulloblastoma cell lines express IL-4R that serve as a sensitive target for IL-4R-targeted agent, cpIL4-PE. Medulloblastoma cell lines were found to over-express IL-4Rα and IL-13Rα1 chains. We have previously shown that a large number (41 of 46 samples) of glioblastoma biopsy samples or primary cell cultures are positive for IL-4Rα and IL-13Rα1 chains as determined by RT–PCR and immunofluorescence analyses (Joshi *et al*, 2000; Puri *et al*, 1996a). Our present results confirm those observations and demonstrate that medulloblastoma cell lines also express IL-4Rα and IL-13Rα1 chains in all cell lines tested. The absence of γ_c_ protein indicated that medulloblastoma cell lines express type II IL-4R as do malignant glioma cells. This configuration is similar to that observed on other tumour cells including renal cell carcinoma, AIDS-Kaposi's tumours, head and neck tumours, colon carcinoma, ovarian carcinoma and breast tumour cells (Husain *et al*, 1997; Kawakami *et al*, 2000; Kreitman *et al*, 1994; Leland *et al*, 2000; Murata *et al*, 1997b; Puri *et al*, 1996b).

It is intriguing to note that D283 cell line expressed both chains of IL-4R (IL-4Rα and IL-13Rα1) at the mRNA and protein levels by RT–PCR and indirect immunofluorescence studies, respectively. However, upon direct binding study, it only expressed about 145 IL-4 binding sites/cell. Consistent with low IL-4 binding, this cell line was not sensitive to the cytotoxic activity of cpIL4-PE. The direct binding studies, using ^125^I-IL-4 did not correlate with either immunofluorescent detection of cell surface IL-4/IL-13 receptor subunits or their mRNA transcripts. Thus, these assays may not be able to predict as to which tumours are likely to respond to cytotoxin-based therapy. Therefore, other assays may be required. It is possible that D283 cell line expresses a mutated form of IL-4R, which does not allow binding of IL-4. However, this mutation does not affect its detection by RT–PCR and indirect immunofluorescence assays.

In a recent study, we have observed that one normal brain tissue and single available normal human astrocytic (NHA) primary cell culture expressed mRNA for IL-4Rα and IL-13Rα1 chains (Joshi *et al*, 2000). Upon indirect immunoflorescence studies the NHA primary cell culture was shown to express these chains at the cell surface. In another study, Liu *et al* (2000) have also demonstrated that IL-4R are expressed in normal human astrocytes and glioma cells confirming our initial observations. Thus, it appears that NHA also express type II IL-4R. Additional samples including paediatric brain are being examined to confirm or dispute these observations.

Although expression of IL-4Rα and IL-13Rα1 chains may form a functional IL-4R complex, it is not known whether this receptor complex will internalize the cpIL4-PE after binding. It is also not known whether cpIL4-PE is cytotoxic to normal brain tissues. In our Phase I clinical trial, we did not see any microscopic damage to normal brain tissues in three patients (in which intraoperative biopsy was obtained) that received 0.2, 2 and 6 μg ml^−1^ doses of cpIL4-PE, respectively. In addition, in our recent study, we have found that cpIL4-PE is not cytotoxic or modestly cytotoxic to a neuronal cell line and NHA cell culture compared to 15 glioma primary cell cultures in which cpIL-4PE is highly cytotoxic (Joshi *et al*, unpublished results). Furthermore, it is known that resting B cells, monocytes, bone marrow cells and endothelial cells which express type I or type II IL-4 receptors are not sensitive to cpIL4-PE (Husain *et al*, 1997; Puri *et al*, 1994). These results suggest that there is a large therapeutic window in which one can target cpIL4-PE to cancer cells but cause little collateral damage to normal brain tissues or other immune and non-immune cells. Additional primary normal brain tissues particularly normal cerebellum tissue samples need to be tested for sensitivity to cpIL4-PE.

We have previously observed that none of the solid tumour cell lines including glioma cell lines secrete IL-4. Therefore, IL-4 does not appear to be an autocrine growth factor for tumour cell lines. However, exogenous IL-4 has been shown to mediate contrasting effects on various tumour cell lines. For example, IL-4 can mediate modest inhibition of proliferation of human renal cell carcinoma (Obiri *et al*, 1993), melanoma (Hoon *et al*, 1991), breast and colon carcinoma (Toi *et al*, 1992), and non-small cell lung carcinoma cell lines (Topp *et al*, 1993a, b). On the other hand, IL-4 can modestly stimulate the growth of human squamous cell carcinoma of head and neck cell lines (Myers *et al*, 1996). The reason for contrasting effect of IL-4 is not known. Since, medulloblastoma cell lines did not respond to IL-4, it is reasonable to conclude that IL-4 is not a paracrine growth factor for these cells. To determine the functional activity of IL-4R on tumour cells, we and others have shown that IL-4 can cause phosphorylation of JAK1, JAK2 and STAT6 proteins in human colon carcinoma and ovarian carcinoma cell lines even though IL-4 does not mediate autocrine or paracrine growth of these tumour cell lines (Murata *et al*, 1995, 1997b). These studies suggest that IL-4 induced signal transduction does not seem to mediate growth of tumour cells. Signal transduction may modulate growth inhibition or other unknown biological effects on these cells, which are yet to be discovered. Further studies are required to determine the significance of IL-4R expression on medulloblastoma cell lines.

In conclusion, we report that, like GBM, medulloblastoma cell lines also express IL-4R, which appears to be an efficient target for cpIL4-PE. Additional primary medulloblastoma tumour samples need to be tested for their sensitivity to cpIL4-PE. In addition, the efficacy of cpIL4-PE should be tested in animal models of human medulloblastoma tumours and perhaps in the clinic for the treatment of young children who have failed standard treatment. Alternatively, this agent could be useful in paediatric patients who are most susceptible to the deleterious effects of standard treatment of radiation to developing brain.

## Note added in proof

We have recently reported (Joshi *et al*, 2001) that 15 of 18 glioblastoma mutliforme tumours express IL-4 receptors *in situ*. In contrast, IL-4R protein was not detected by immunohistochemistry in 11 different normal brain tissues including sections from one paediatric brain.
